# Unified modeling of photothermal and photochemical damage

**DOI:** 10.3389/fopht.2024.1408869

**Published:** 2024-08-19

**Authors:** Michael L. Denton, Clifton D. Clark, Gary D. Noojin, Haleigh West, Allison Stadick, Taufiquar Khan

**Affiliations:** ^1^ Bioeffects Division, Air Force Research Lab, JBSA-Fort Sam Houston, TX, United States; ^2^ Department of Physics, Fort Hays State University, Hays, KS, United States; ^3^ Biosciences Department, Science Applications International Corporation, JBSA-Fort Sam Houston, TX, United States; ^4^ Department of Mathematics and Statistics, University of North Carolina at Charlotte, Charlotte, NC, United States; ^5^ Department of Chemistry, University of North Carolina at Charlotte, Charlotte, NC, United States

**Keywords:** laser damage, rate process model, unified model, photothermal, photochemical, Arrhenius, damage integral, thermal profile

## Abstract

Correlating damage outcomes to a retinal laser exposure is critical for diagnosis and choosing appropriate treatment modalities. Therefore, it is important to understand the causal relationships between laser parameters, such as wavelength, power density, and length of exposure, and any resulting injury. Differentiating photothermal from photochemical processes in an *in vitro* retinal model using cultured retinal pigment epithelial cells would be a first step in achieving this goal. The first-order rate constant of Arrhenius has been used for decades to approximate cellular thermal damage. A modification of this equation, called the damage integral (Ω), has been used extensively to predict the accumulation of laser damage from photothermal inactivation of critical cellular proteins. Damage from photochemical processes is less well studied and most models have not been verified because they require quantification of one or more uncharacterized chemical species. Additionally, few reports on photochemical damage report temperature history, measured or simulated. We used simulated threshold temperatures from a previous *in vitro* study to distinguish between photothermal and photochemical processes. Assuming purely photochemical processes also inactivate critical cellular proteins, we report the use of a photothermal Ω and a photochemical Ω that work in tandem to indicate overall damage accumulation. The combined damage integral (Ω_CDI_) applies a mathematical switch designed to describe photochemical damage relative to wavelength and rate of photon delivery. Although only tested in an *in vitro* model, this approach may transition to predict damage at the mammalian retina.

## 1 Introduction

The retina is a primary target organ for laser damage. The pigmentation of the retinal pigment epithelial (RPE) layer efficiently absorbs and converts visible (VIS) and near-infrared (NIR) light into heat, which can damage cells and tissues by photothermal inactivation of critical cellular proteins. Photons with sufficient energy to produce photooxidative stress (short visible) pose an additional threat to the RPE. This dual threat creates safety issues for some retinal applications of lasers in the blue spectrum, such as autofluorescence imaging (1, 2). Therefore, correlating damage outcomes to a retinal laser exposure is critical for diagnosis and choosing appropriate treatment modalities.

Investigations into predicting photothermal (laser) damage found a correlation to the combination of temperature and time (temperature history). This led laser researchers to use the adaptation to the temperature dependent Arrhenius first-order rate constant (3) developed by Enrique and Moritz (4–6), called the damage integral (Ω),


(1) $$\text{Ω}\;=\;{\int_{0}^{\tau }Ae}^{-\frac{E_{a}}{{RT}_{\left(t\right)}}}\;dt,$$

where A is the frequency factor (s^-1^), E_a_ is the activation energy (J mole^-1^), R is the universal gas constant (8.31 J mol^-1^ K^-1^), and T(t) is the temperature (K) at each time step. Damage accumulates throughout the exposure duration (τ). Current convention is that damage to a cell occurs when Ω reaches a value of unity (1). The integration of temperature versus time data of an exposure (thermal profile), with the trigger at Ω=1, is a common metric for determining if an exposure was or is expected to be damaging. Accurate assessment and prediction of damage requires correct Arrhenius parameters (A and E_a_), which are obtained empirically using the Arrhenius plot (Ln (τ) vs inverse peak temperature (K^-1^)). Several Arrhenius parameter pairs (A/E_a_) have been reported for various tissue types and cultured cells (7).

Modeling photochemical damage would seem more complex than photothermal damage. Any detailed mechanistic assessment for photochemical cellular damage must include the production of reactive oxygen and nitrogen species (RXS) leading to oxidative stress (8, 9). Models using rates of formation of compounds requires knowledge of the chemical reactants and products (and their concentrations), as well as the kinetic rates for each reaction. Products can be small molecule RXS or adducts associated with biochemicals, such as lipid peroxides and protein reactive species (10–14). Often, the original photon acceptor (chromophore) and the individual chemical species involved are not known (15, 16). It is difficult to test such models.

Like all cells, RPE cells are susceptible to photochemical damage from exposure to short visible wavelengths (17), often termed the blue light hazard (18). Their anatomical location and function in the vision cycle place RPE cells in an environment rich in oxygen and polyunsaturated fatty acids. When combined with routine exposure to blue light, these factors produce long-term oxidative stress thought to be responsible for age-related macular degeneration (AMD) (17). Thus, retinal irradiation with blue laser light adds an acute level of damage processes to the ongoing long-term metabolic stress of the RPE cells. Specifically, light interacting with melanin and lipofuscin, which act as photosensitizers, may present an elevated threat of oxidative stress to RPE cells in particular (17, 19–21).

Regardless of photooxidative mechanisms, purely photochemical processes are expected to follow the rule of irradiance (E, J s^-1^ cm^-2^) reciprocity. The principles of the Bunsen-Roscoe law of reciprocity (22), and later conveyed by Dworkin (23), states that the extent of photochemical reactions is proportional to total photon dose in radiant exposure (H, J cm^-2^). Thus, if exposure duration is doubled, threshold irradiance for the effect is reduced by a factor of two (E x τ), leading to the same threshold radiant exposure. We exploit this relationship in our modeling efforts described here.

Our initial model to predict the transition from photothermal to purely photochemical damage (16) was implicit and required a value for concentration of an unknown oxidative product (B^*^). That study concluded with a mathematical expression that predicted the transition but follow up work was recommended. Here, we simplify the modeling process with the assumption that the photochemical rate process results in the inactivation of critical cellular proteins via oxidative reactions with RXS. With this premise, we merged the well-known photothermal Ω (Ω_PT_) with a novel photochemical Ω (Ω_PC_) to develop the combined damage integral (Ω_CDI_). Using a step function switch based on an empirically determined threshold in photon flux (ϕ_p_) and simulated threshold *in vitro* thermal profiles at 413 nm we assessed the accuracy of the Ω_CDI_ model.

## 2 Combined damage rate process model

Our primary hypothesis is that damage from photooxidation is a result of inactivation of one or more key cellular proteins, likely enzymes. This hypothesis is congruent with the proposed process for thermal and photothermal damage. This concept led to the idea of combining the individual damage integrals into a single one (Ω_CDI_) and, using the same thermal profiles, the two should solve for unity at threshold damage.


(2) $${\text{Ω}}_{CDI}={\int_{0}^{\tau }A_{PT}e}^{-\frac{E_{a}^{PT}}{{RT}_{\left(t\right)}}}\;dt+{\int_{0}^{\tau }A_{PC}e}^{-\frac{E_{a}^{PC}}{{RT}_{\left(t\right)}}}\;dt={\text{Ω}}_{PT}+{\text{Ω}}_{PC}$$

In Equation 2, ${\text{A}}_{\text{PT}}$ and ${\text{E}}_{\text{a}}^{\text{PT}}$ represent the frequency factor and activation energy for photothermal damage, respectively. Alternatively, the frequency factor and activation energy for purely photochemical damage is ${\text{A}}_{\text{PC}}$ and ${\text{E}}_{\text{a}}^{\text{PC}}$ , respectively. Notice that temperature is a universal switch for the damage integrals due to its location in the negative exponent. When thermal energy, in the form of system temperature, becomes great enough to overcome the energy barrier (E_a_) the exponent term becomes a smaller negative value and the magnitude of the overall expression increases.

**Figure 1:**
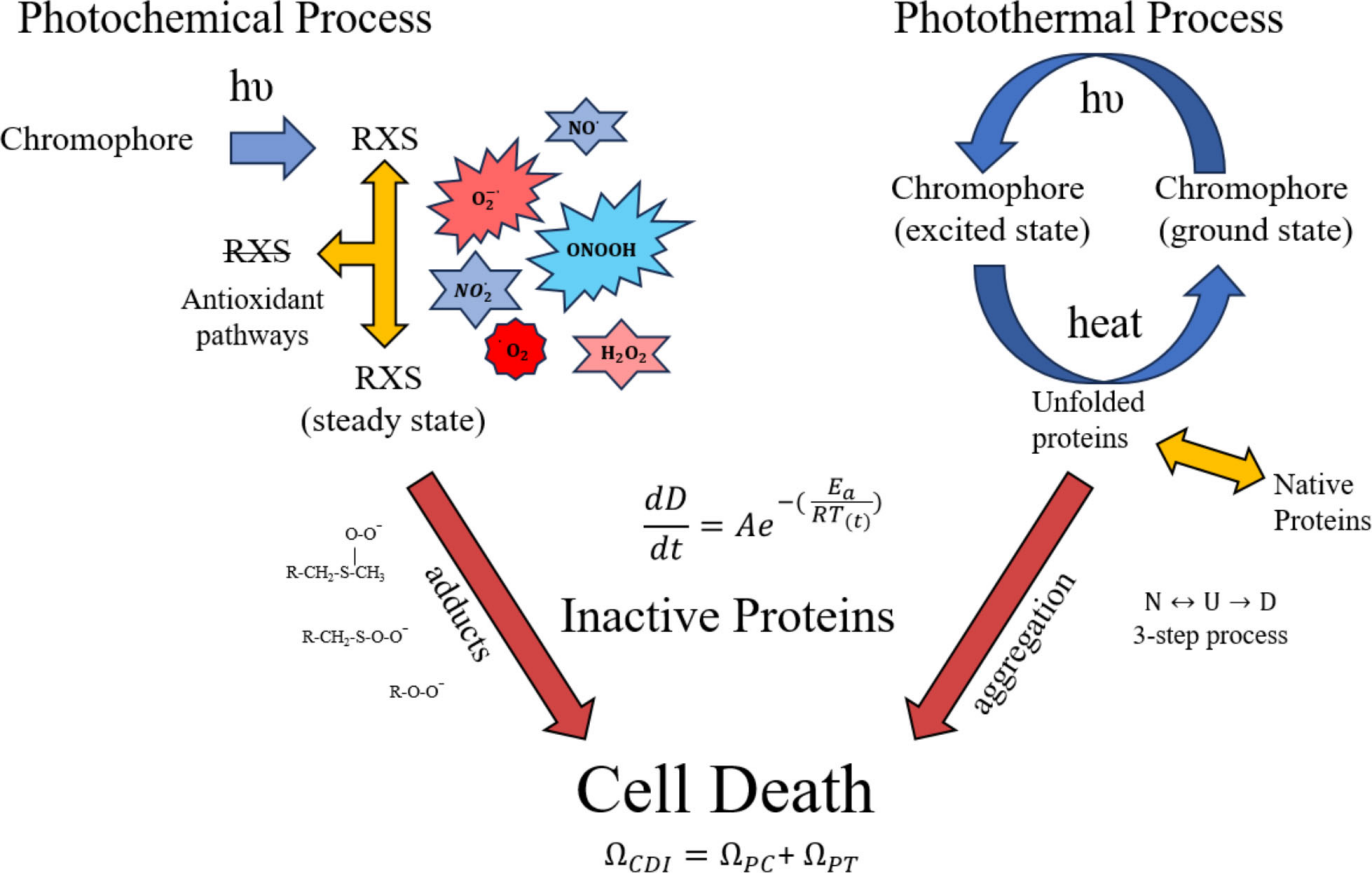
Proposed processes for photothermal and photochemical damage. Once photon absorption in photooxidative chromophores generates RXS, some are removed by cell antioxidant mechanisms while others participate in inactivating proteins as oxygen/nitrogen adducts. The heat generated from non-radiative decay of photon absorption (photothermal) leads to unfolding and aggregation of proteins, thus inactivating them. The differential equation (change in damage per unit time) indicates an instantaneous rate of protein inactivation and Ω_CDI_ is the damage accumulation rate. The 3-step protein denaturation equation shows a reversible transition between native (N) and unfolded (U) protein, and the irreversible step of aggregation/denaturation (D). Primary amino acids targeted for oxidation are methionine and cysteine. A few of the possible RXS and protein adducts are provided for illustration purposes. Red arrows indicate irreversible processes of protein oxidation and aggregation.

**Figure 1** describes the combined damage rate hypothesis in graphical form. The initial step in the photothermal process is photon-driven excitation of electrons with subsequent internal conversion and vibrational relaxation that generates heat. The rate of heating depends on photon absorption driven by laser irradiance and the optical and thermal properties of the tissue being exposed. The heat facilitates reversible unfolding of macromolecules, including proteins. At temperatures below damaging levels, proteins can refold, often with the aid of chaperone proteins called heat shock proteins. When enough proteins are unfolded, they aggregate and become irreversibly denatured (24, 25), as depicted by the red arrow in **Figure 1**. Thus, the instantaneous rate of damage (differential equation in **Figure 1**) is laser irradiance dependent, and the accumulation of damaged proteins can be determined by the damage integral for the photothermal process (Equation 2).

Although details of the photochemical process are less well known, we have approximated the processes to include antioxidant activity. The antioxidant activity, which is likely dependent upon the reduction/oxidation (RedOx) state of the cell, is responsible for steady state levels of RXS. One would expect a greater steady state concentration of RXS produced as laser irradiance is increased, assuming photon energy is sufficient for the photooxidation reaction. As laser irradiance increases for a given exposure duration, the steady state concentration of RXS increases and antioxidant activity becomes overwhelmed. For extended exposure durations there may be compensatory antioxidant activity that must be overcome by the minimal amount of photooxidation needed to generate threshold damage. In this fashion, laser irradiance dictates steady state levels of RXS while time of exposure dictates the accumulation of damaged proteins via chemical adducts with amino acids, predominantly methionine and cysteine (12). A simplistic description of irradiance reciprocity would be the combination of a steady state concentration of RXS (irradiance), and the number of protein oxidative hits achieved during the exposure (radiant exposure). Analogous to the photothermal scenario, the instantaneous damage rate depends upon the steady state level of RXS (irradiance) and the accumulation of damaged proteins can be determined by the damage integral for the photochemical process (Equation 2). Like the irreversible aggregation of proteins in the photothermal process there is little reversal of protein oxidation, so the only negating factor for photochemical damage would be the antioxidant activity and any compensatory shift in response to the photooxidation.

When photon energy is sufficient to generate both heat and RXS, such as in pigmented RPE cells, the potential of concurrent photothermal and photochemical processes must be considered. Both processes are driven by irradiance and time of exposure, but only purely photochemical processes follow irradiance reciprocity. One way to reconcile an overall mixed damage process is to correlate the instantaneous rates for inactivation of proteins for the two processes described in **Figure 1**. A simplistic view is that the instantaneous rates of protein inactivation ( $\frac{dD}{dt}$ ) vary differently with laser irradiance for each process. At higher irradiances, the rate of thermal inactivation exceeds the rate for photochemical inactivation, and elevation above a certain temperature is expected to inhibit the photochemical process. However, up to this inactivation temperature, which is currently unknown, there would be an increase in photochemical oxidation as temperature is elevated. At lower irradiances photothermal processes are minimized in a temperature dependent manner, leaving the way for photochemical damage, again depending upon irradiance, wavelength, and exposure duration.

**Figure 2:**
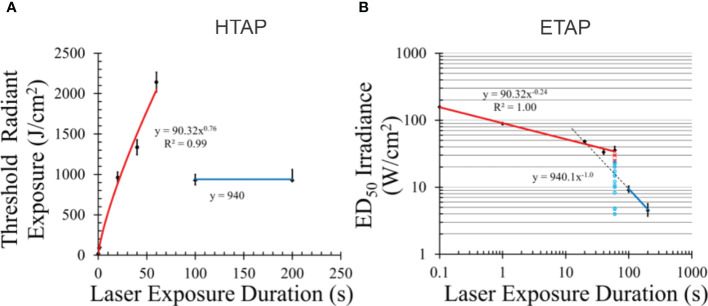
Temporal action profiles (TAP) for damage to pigmented hTERT-RPE1 cells at 413 nm. Radiant exposure **(A)** and irradiance TAP **(B)**. Error bars represent one standard deviation. Blue lines indicate irradiance reciprocity between 100 and 200 s exposures (purely photochemical). Red lines represent damage with some or all photothermal component. Dashed line in **(B)** is an extrapolated line using the principle of irradiance reciprocity. Pertinent raw data for 60 s are indicated as damaged (red x) and undamaged (aqua circles) in **(B)**. Power functions for each line are given. [Figures adapted from Denton et al. (26) and Denton et al. (27)].

The Ω_CDI_ model needs some form of photooxidative switch to indicate when the system transitions to purely photochemical damage. The switch must distinguish damage mechanisms when the same number of photons are delivered rapidly versus slowly. This time dependence is described in the next section (**Figure 2**).

### 2.1 Data used to test the CDI model

#### 2.1.1 Threshold damage

The CDI model requires threshold thermal profiles for laser exposures across a broad enough range of durations to transition from purely photothermal to purely photochemical damage. No complete set of empirical thermal data is currently available, even for *in vitro* systems. Previously, we published Probit threshold irradiance ED_50_ values for 413-nm exposures (0.3-mm diameter) in an artificially pigmented *in vitro* retinal model based on hTERT-RPE1 cells (26). **Table 1** provides the threshold irradiance and radiant exposure values for that published study. The range of exposure durations was from 0.1 to 200 s. The table also provides simulated peak thermal responses using the ED_50_ irradiance values (see below).

**Table 1:** Threshold damage parameters for *in vitro* laser exposures at 413 nm.

	E	H	Sim. Peak T	Sim. Peak ΔT
τ (s)	(W cm^-2^)	(J cm^-2^)	(°C)	(°C)
0.1	157	15.7	63.0	28.0
1	88.7	88.7	59.7	24.7
20	48.1	962	51.9	16.9
40	33.4	1336	47.0	12.0
60	35.7	2142	48.0	13.0
100	9.4	940	38.4	3.4
200	4.7	940	36.7	1.7

Exposure duration (τ). Threshold irradiance (E). Threshold radiant exposure (H). Simulated peak temperature (Sim. Peak T) and temperature rise (Sim. Peak ΔT). Simulated thermal profiles are found in **Figure 4**. [Some data taken from Denton, et al. (26)].

A common method of expressing laser threshold damage is the temporal action profile (TAP), where threshold radiant exposure (HTAP) or irradiance (ETAP) is plotted versus exposure duration. **Figure 2** provides the HTAP and ETAP graphs for the 413-nm data in **Table 1**. Each TAP analysis shows interesting trends, especially when laser wavelength supports the generation of purely photochemical damage as shown for 413 nm. As our previous paper describes (26), the transition from photothermal to purely photochemical damage has been reported for *in vivo* models as well as our *in vitro* retinal model. Thus, understanding this transition in damage mechanisms *in vitro* will begin to address data gaps in the current (2022) American National Standards Institute (ANSI) Z.136.1 Standard. Currently, photothermal and photochemical effects are treated as independent and described as dual limits. Under this premise, maximum permissible exposures (MPEs) are calculated independently, and the most restrictive limit is used. This implies no synergy or additivity of damage processes.

Salient features of the HTAP (**Figure 2A**) include a line (blue) with a slope of zero, which represents irradiance reciprocity at 940 J cm^-2^, which is also indicated in the ETAP (**Figure 2B**) as the blue line with a power function with a slope of -1.0 (reciprocity). From this analysis, damage at 100 and 200 s for these 413-nm exposures was purely photochemical. The red lines in both TAP graphs represent damage with some unquantified degree of thermal component, whether purely photothermal or mixed photothermal and photochemical. Temperature data for these exposures, whether recorded during laser exposure or simulated using computational models, would provide evidence of thermal component. Below, we show simulated temperature rises from the threshold irradiances.

Radiant exposure, in terms of J cm^-2^, is a measure of the number of photons delivered per unit area for a given wavelength. Thus, the H threshold for purely photochemical damage is correlated with the number of photons delivered, per area, and wavelength. In both TAP graphs, extrapolation of the purely photochemical damage line crosses the photothermal line at around the 20-s data point. If data were collected at only 20, 100, and 200 s, it would have indicated irradiance reciprocity extending down to 20 s. However, the 20-s data clearly lies on the photothermal line even though the same number of photons per area were delivered. Obviously, the threshold metric for photochemical damage cannot be based on the number of photons delivered per unit area. The threshold H values at 20, 40, and 60 s were similar or greater than the 100-s and 200-s thresholds (**Table 1**) and are still on the thermal trendline. Clearly, greater than 940 J cm^-2^ were delivered in a shorter time during the 20 – 60 s exposures than for the 100 and 200-s thresholds. **Figure 2B** provides raw damage data, where red x’s and aqua circles indicate damaged and nondamaged outcomes, respectively. If there were some photochemical injuries in addition to photothermal damage generated at the 60-s duration, they would have manifested in red x’s at the lower irradiances. Overall, these discontinuities must be addressed in the CDI model.

#### 2.1.2 Threshold temperatures

**Figure 3:**
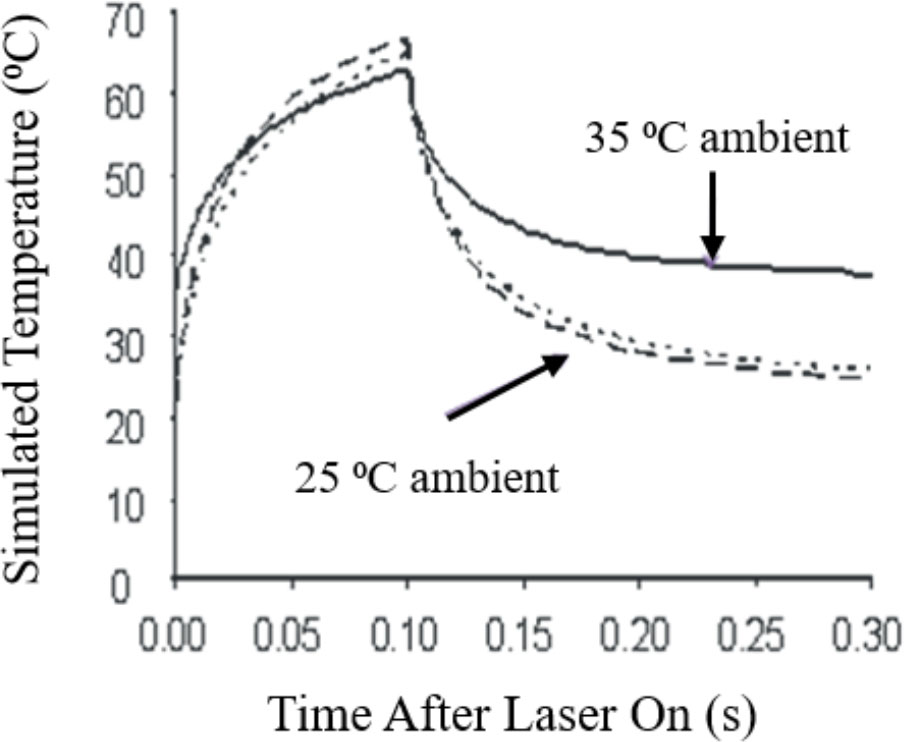
Computational simulations of 0.1-s *in vitro* laser exposures. Short-dashed line: 25°C ambient, 413 nm, 0.2-mm buffer, ED_50_ = 334 W/cm^2^. Long-dashed line: 25°C ambient, 532 nm, 3.0-mm buffer, ED_50_ = 664 W/cm^2^. Solid line: 35°C ambient, 413 nm, 0.2-mm buffer, ED_50_ = 157 W/cm^2^.

To characterize the thermal component for the damage data represented in **Figure 2** we simulated temperature rises using the BTEC thermal model (28). By simulating the maximum temperature (single pixel equivalent) at the center of the exposure using threshold irradiances (**Table 1**), similar to a minimum visible lesion (MVL), the resulting thermal profiles are considered threshold thermal responses. **Figure 3** illustrates the consistency of the simulation method for a purely photothermal condition (0.1 s). The three thermal profiles shown were simulated from three different combinations of laser wavelength, threshold irradiance, ambient temperature, and the height of buffer covering the artificially pigmented RPE cultures. Pigmentation was held constant over the three examples. On average, the area under the thermal profiles from 0.0-0.1 s are similar and would indicate good approximation of the thermal requirement for killing the RPE cells, regardless of the variables in laser parameters and sample boundaries.

**Figure 4:**
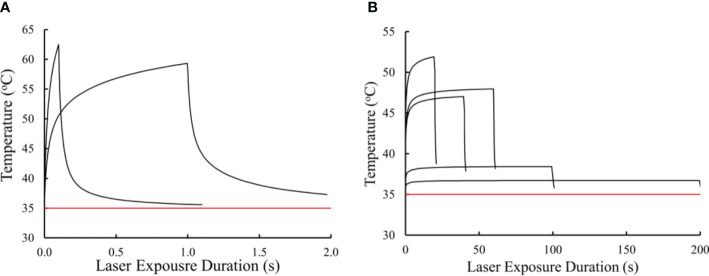
Simulated *in vitro* thermal responses to threshold laser irradiance at 413 nm. Thermal profiles for 0.1 and 1.0 s **(A)**. Thermal profiles for 20, 40, 60, 100, and 200 s **(B)**. Red line represents ambient temperature (35 °C).

**Figure 4** presents the thermal profiles generated for the data in **Table 1**. The profiles represent laser exposures of 0.1, 1.0, 20, 40, 60, 100, and 200 s at 413 nm. Exposures of 40 s and longer reached steady state temperatures. Graphically, it appears the 20-s exposure did not achieve steady state temperature. Due to differences in irradiance, the 60-s irradiance led to a slightly higher simulated temperature than that of the 40-s exposure (**Table 1**). This difference is likely not significant due to the uncertainty of the thermal camera (± 1°C) and the 12% uncertainty in the measurement of laser irradiance. This data implies similar damage rate processes for exposures of 40 and 60 s. If there are mixed damage mechanisms occurring at 40 and 60 s, the ratios between photothermal and photochemical is expected to be similar. **Table 1** also provides simulated threshold peak temperatures (at the end of τ) for the 413-nm exposures. These thermal profiles have not been published previously.

Equipped with the simulated thermal profiles we can begin to ascribe thermal characteristics to damage processes at the different exposure durations. For instance, the temperature rise for the two shortest exposures were greater than 20° C, indicating purely photothermal damage processes. Due to irradiance reciprocity the 100-s and 200-s exposures showed reciprocity for temperature rise (3.4 and 1.7 °C, respectively). Interestingly, the temperature rises from exposures of 20, 40, and 60 s were all between 10 and 20 °C. It is generally accepted that temperature rises above 10 °C are indicative of thermal damage (29), while others have stated the belief that temperature rises in the range of 10 – 20 °C are likely a combination of thermal and photochemical damage processes (30). Using these thermal profiles, we will use the CDI model to predict the transition to purely photochemical damage processes between the 60 and 100 s exposures.

#### 2.1.3 Arrhenius plot

**Figure 5:**
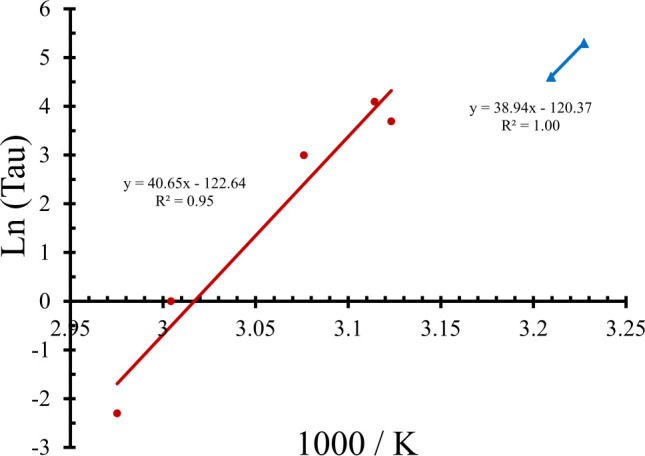
Arrhenius plot using peak temperature (end of τ) and exposure duration data from **Table 1**. Value of E_a_ is obtained by multiplying the Arrhenius plot slope (after factoring the true value of K) by the ideal gas constant (8.31 J mol^-1^ K^-1^). The antilog (base e) of the negative y-intercept value gives the Arrhenius A value.

In order to identify differences and similarities in the 0.1 – 60 s versus the 100 – 200-s exposure damage rate processes we plotted the Ln(τ) and inverse peak temperatures (K^-1^) as an Arrhenius plot (**Figure 5**). We found similar values between the two groups for E_a_ (from slope) and A (from y-intercept). Obviously, the line generating the Arrhenius E_a_/A pair from the shorter exposures was an average of the five data points, with a correlation coefficient of 0.95.

The activation energy from the photothermal and photochemical lines were 337,980 and 323,731 J mol^-1^, respectively. The Arrhenius A values for the photothermal and purely photochemical lines were 1.83 x 10^53^ and 1.89 x 10^52^ s^-1^, respectively. These similar values for the two processes support our hypothesis that both damage processes involve the same mechanisms, relating back to an inactivation of important intracellular macromolecules such as proteins. These Arrhenius rate parameters will be used when integrating the thermal profiles for the respective photothermal (Ω_PT_) and purely photochemical (Ω_PC_) damage integrals.

#### 2.1.4 Threshold average photon flux as a mathematical switch

Two unresolved challenges with the CDI model become evident when referring to Equation 2 and the HTAP in **Figure 2**. As pointed out in Section 2.1.1, the rate of photon delivery is an important feature determining whether damage occurs purely by photochemical processes (100-200 s) or one that has some degree of photothermal processes (0.1-60 s). This is exemplified by the fact that the same radiant exposure was delivered in exposures of 20, 100, and 200 s, but the 20-s irradiance was not in reciprocity with the 100-200-s exposures. The second challenge is that, by nature of Equation 2, a significant Ω_PC_ contribution to Ω_CDI_ would occur when integrating the significant temperatures from the 0.1-60-s thermal profiles. The CDI model needs to account for these discrepancies, but in a manner that is not entirely determined by the empirical quantity defined by Ω. To do this, a mathematical switch, defined by Equation 3, is used to modify the Arrhenius A factor of Ω_PC_.

Although radiant exposure is a convenient expression of both irradiance and exposure duration, it must be ruled out as a switch due to the nonlinear relationship between H and damage mechanism previously described. A switch based solely on laser irradiance does not account for differences in laser wavelength and tissue absorption, such as variable pigmentation in RPE cells and the lack of pigment in most other cells. Although exact photochemical chromophores and chemical species are not known, a known chemical feature is the requirement for a minimum photon energy. Photons from light with wavelengths longer than around 514 nm have not been shown to generate damage at low irradiances, long exposure durations, and low temperatures (30). Thus, wavelength or frequency of the light should be used as part of the photochemical switch function.

Initially, we considered converting H to photon flux density by dividing by the energy of the photons (E_p_) at a given wavelength. Of course, threshold flux density versus exposure duration produced the same plot as the HTAP. However, when converting photon flux density to photon flux ( ϕ ), using the area of the laser beam, we get a graph similar to the ETAP. Threshold irradiance could be used to generate an instantaneous ϕ value, but this metric would not account for exposures with variable irradiance, or the time interval for the delivery of the photons. To distinguish between instantaneous photon flux from that delivered in 20 s and 100 s, we enlisted a “tau factor” (inverse exposure duration) with the threshold radiant exposure to produce the average photon flux delivered over the course of τ ( $\phi_{\tau }$ ), as shown in Equation 3.


(3) $$\phi_{\tau }=\frac{photons}{s}=\;\frac{H^{thr}\times \;A_{L}}{\left(\upsilon \times h\right)}\;\times \;\frac{1}{\tau }$$

Here, H^thr^ is threshold radiant exposure (J cm^-2^), A_L_ is area of the laser beam (cm^2^), υ is laser frequency (s^-1^), and h is Plank’s constant (6.63 x 10^-34^ J s). The product (υ×h) defines E_p_ in joules. Thus, the calculation for $\phi_{\tau }$ uses multiple laser parameters and physics concepts. The $\phi_{\tau }$ switch considers that purely photochemical damage requires a given ϕ be delivered over a specified time. This deconvolves the problem with the HTAP analysis by simplifying to the average rate of delivery of photons. It also supports the hypothesis that threshold average irradiance produces just enough steady state RXS molecules to overcome repair mechanisms in the shortest τ possible to generate photochemical damage. From this analysis, $\phi_{\tau }$ is our metric for the mathematical switch in the CDI model.

**Figure 6:**
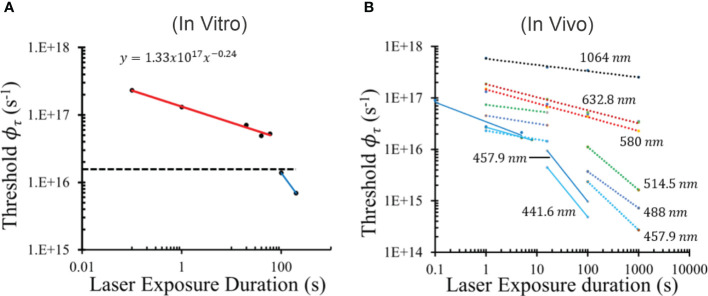
Threshold average photon flux shows a defined break point between damage mechanisms. The graph of threshold photon flux provides a break point between the purely photochemical damage and the photothermal and mixed mechanism damage. *In vitro* threshold photon flux at 413 nm versus τ **(A)** shows the ϕ_τ_ value used for the CDI model photochemical switch function (dashed black line, 1.4 x 10^16^ photons s^-1^). *In vivo* threshold ϕ_τ_ values plotted versus τ **(B)** using data reported by Lund (31) (solid lines) and Ham (30) (dashed lines). Line colors and annotations indicate wavelengths.


We have converted threshold radiant exposure from **Table 1** to threshold $\phi_{\tau }$ (A_L_ = 0.3 mm) and plotted them versus exposure duration (**Figure 6A**). Like the ETAP graph, the threshold $\phi_{\tau }$ graph has a trendline for the 0.1 – 60-s exposures, while the 100 and 200-s trendline shows reciprocity (τ^-1^, slope not shown). Similar to both TAP analyses, the wide gap in photon flux between the 60-s and 100-s exposures makes it difficult to know in greater temporal resolution where and how steep is the transition to purely photochemical damage. For this reason, a value of 1.4 x10^16^ s^-1^ (**Figure 6A** dashed line) was chosen for the mathematical threshold $\phi_{\tau }$ switch function in Ω_PC_, as shown in Equation 4.

(4) $${\text{Ω}}_{CDI}=\;\int_{0}^{\tau }A_{PT}e^{-\frac{E_{a}^{PT}}{RT\left(t\right)}}\;dt+\int_{0}^{\tau }(\chi \left[\phi_{thr}-\phi_{\tau }\right]\times A_{PC})e^{-\frac{E_{a}^{PC}}{RT\left(t\right)}}\;dt$$

Here, the characteristic function χ (y) is 0 if y is negative and is 1 if y is non-negative. This step function defines the mathematical switch for progression from some thermal component to purely photochemical damage. If and when more data is collected to determine the shape of the transition curve from photothermal to purely photochemical damage processes, we can implement the tanh function to indicate the gradual transition.

#### 2.1.5 Combined damage integral values

Confirmation that the CDI model functions properly is established by integrating the thermal profiles in **Figure 4** with Equation 4, using the Arrhenius rate constants obtained from **Figure 5**. **Table 2** provides the results for Ω_CDI_, Ω_PT_, and Ω_PC_. As a measure of accuracy, percent deviations of Ω_CDI_ values from Ω=1 are also shown in **Table 2**. The CDI model was most accurate for exposures of 60-200 s. Surprisingly, the Arrhenius rate constants for Ω_PT_ did not indicate purely photothermal damage processes at the shortest exposure durations but was accurate for the 60-s thermal profile.

**Table 2:** Combined damage integral values for *in vitro* 413-nm simulated thermal profiles.

τ (s)	Ω_CDI_	Ω_PT_	Ω_PC_	% Difference
0.1	0.14	0.14	0.00	86
1	0.76	0.76	0.00	24
20	1.37	1.37	0.00	37
40	0.45	0.45	0.00	55
60	0.99	0.99	0.00	1
100	1.03	0.04	0.99	3
200	1.05`	0.04	1.01	4

Method groups damage from 0.1 – 60-s exposures separately from purely photochemical at 100 – 200 s. Percent difference from unity in Ω_CDI_ values is given.

With the photochemical switch in place, the values of Ω_PC_ for exposure durations shorter than 60 s were zero. In addition, the relatively low temperatures of the 100-200-s thermal profiles reduced Ω_PT_ to essentially zero, while the same profiles contributed to Ω_PC_=1. This remarkable result suggests the A/E_a_ values for the two damage processes, although similar, were well suited for exposure durations spanning the sharp transition in damage mechanisms. The result also supports the use of separate Arrhenius plots for the purely photochemical, and partially or all thermal damage processes in our model.

The use of $\phi_{\tau }$ as a photochemical switch function works well within the limited scope of the simulated 413-nm thermal profiles from *in vitro* exposures. To assess the power of the method when applied to *in vivo* models, damage threshold radiant exposures for a wide range of laser wavelengths reported in the literature (30, 31) were converted to $\phi_{\tau }$ and plotted in **Figure 6B**. These data show a distinct wavelength dependence for the transition to photochemical damage occurring in wavelengths of 514 nm and shorter. There was also a large gap between the trendlines that do break from “photothermal” to “photochemical” damage processes. Here, the authors did not optimize for identifying the sharp transition in mechanisms because their studies were not designed to address this issue. For this reason, none of the “photochemical” lines showed irradiance reciprocity (slopes were not τ^-1^). The power function slopes for “photochemical” lines of 441.6 nm and 457.9 nm of Lund (16 s, 100 s) were -1.24 and -1.21, respectively. Slopes for the “photochemical” lines of 457.9 nm, 488 nm, and 514.5 nm data of Ham (100 s, 1,000 s) were -0.94, -0.71, and -0.84, respectively. These results do indicate that, for these *in vivo* models, there can exist some degree of photothermal processes after the $\phi_{\tau }$ break point and more data is required to spatially resolve the transition in damage mechanisms.

It should be noted that the method for determining threshold radiant exposure differs for the groups of Lund and Ham. The Probit method (both Lund and Denton) uses a probability function to determine the irradiance (and thus radiant exposure) value that would cause some sort of damage (large or small) 50% of the time for a given set of experimental conditions. Ham’s group uses the lowest value that generates a damage outcome and is therefore always a lower threshold value relative to Probit thresholds. This may account for some of the differences in the data shown in **Figure 6B**. Regardless, there appears to be a universal level of $\phi_{\tau }$ in the *in vivo* data that represents a potential photochemical switch function. Using the largest $\phi_{\tau }$ value representing photochemical damage (100-s 514.5-nm data point in **Figure 6B**), a possible *in vivo* threshold $\phi_{\tau }$ switch occurs at around 1.4 x 10^16^ photons s^-1^ for all the *in vivo* data shown. Even though this value is identical to the value chosen for the *in vitro* data, the *in vivo* switch point may differ based on magnified uncertainties associated with laser irradiance and area estimated at the retina. These values are more accurately measured in *in vitro* models. Overall, **Figure 6** indicates that our proposed photochemical switch metric might be applied universally.

## 3 Summary and discussion

The RPE layer is sensitive to both photothermal and photochemical damage processes from short visible laser exposure due to the presence of melanosome particles. Understanding any interplays between the processes, such as additivity or synergy for damage overall, is a particularly important consideration. Currently, the ANSI Z136.1 standard (32) uses a dual limit approach when determining retinal MPEs for exposure wavelengths within the blue light hazard region. These independent calculations imply no synergy or additivity of damage processes. However, simulated temperature rises for purely photochemical damage at 413 nm was 3.4°C (**Table 1**) and Ham *et. al* (30). suggested combined retinal damage mechanisms when exposed to blue wavelengths at low power. Also, it is becoming clear that purely photochemical damage can be accelerated by elevated temperatures (33). These potential unknown retinal hazards are likely not considered for clinical assessments of RPE health using blue lasers, such as lipofuscin autofluorescence (1, 2, 34, 35).

Laser damage experiments cannot be performed in humans, so predicting retinal damage using computational models is an important supplement to data collected from *in vivo* and *in vitro* models. Thus, effective computational models predicting damage based on laser wavelength, irradiance, exposure duration, and thermal responses are needed. Understanding how photon interactions inactivate biomolecules is the mechanistic way to devise good models. Conversely, novel computational models based on a mechanistic premise can aide in our understanding of underlying biochemical processes.

This paper tests our hypothesis that photothermal and photochemical damage processes are similar, allowing the adoption of similar mathematical principles. Predictive thermal damage models based on the Arrhenius second order rate constant have been successfully used for decades. The damage accumulation model, called the damage integral Equation 1, expresses dependence on both temperature and time. The Ω uses an exponential mathematical switch with the ratio of temperature to the activation energy for the overall damage process. Mainstream dogma for thermal damage correlates E_a_ to the energy required to irreversibly inactivate (denature) proteins. In order to predict damage from laser exposure, the Ω requires a thermal history in the form of a thermal profile. For use in this paper, thermal profiles were simulated using published Probit ED_50_ threshold laser doses for 413-nm exposures in our *in vitro* retinal model (36). These *in vitro* damage thresholds show a rapid transition in mechanism to purely photochemical in a manner consistent with *in vivo* data in the literature.

Photooxidation of proteins also irreversibly inactivates them, so we performed separate Arrhenius plots (**Figure 5**) for the 0.1-60-s exposures (some thermal component) and the 100-s and 200-s exposures. The resulting Arrhenius rate coefficients A and E_a_ were similar between the two groups, indicating similar damage processes. If the processes for photothermal and photochemical damage are the same, we should be able to predict damage as a combined damage integral (Ω_CDI_), as given in Equation 2. The Ω_PT_ and Ω_PC_ components would each have their own Arrhenius A/E_a_ pair based on the Arrhenius plot (**Figure 5**). The only confounding factor was how to eliminate the elevation in Ω_PC_ value in the exposures generating significant temperature rises (0.1-60 s). Without full knowledge of the temporal transition from photothermal to purely photochemical processes between 60 and 100 s, we elected to implement a mathematical step function using the calculated average photon flux at the end of τ Equation 3. The $\phi_{\tau }$ switch is based on the threshold $\phi_{\tau }$ value for the 100-s exposure, well below the threshold $\phi_{\tau }$ value for the 60-s exposure.

The final mathematical model for Ω_CDI_ is shown in Equation 4. **Table 2** summarizes the results of integrating the thermal profiles (**Figure 4**) using Equation 4. The CDI model worked very well for the 60-200-s exposures, where the Ω_PT_ was either near one (60 s) or zero (100 s, 200 s), and the Ω_PC_ was near zero (60 s) or one (100 s, 200 s). The photochemical switch described in Equation 4 worked universally for exposures shorter than 100 s. Expected deviations from the ideal case (Ω_CDI_ =1) are shown in **Table 2**. Oddly, the A/E_a_ pair for the thermal component trendline had the worst performance for predicting photothermal damage from the 0.1-s and 1.0-s exposures, which were expected to be purely photothermal.

We expected any deviation of Ω_CDI_ for the intermediate exposure durations (20-60 s) would indicate a scenario where the damage process was mixed, with a progression towards photochemical as τ was extended. However, there was no trend in the performance of the model relative to exposure duration, and the result of the Ω_CDI_ for 60 s was a perfect fit. This result was surprising when considering steady state temperatures of the 40 s and 60 s were indistinguishable (**Figure 4**) and the overall thermal dose was greater for the 60 s (temperature x time equivalent).

Overall, the percent differences of the Ω_CDI_ values in **Table 2** are in line with those previously reported for 2-µm laser exposures in our *in vitro* retinal model (37), where the range of Ω_PT_ was 70-155% from unity for 0.1-20-s exposures. Considering the expected damage process for 2-µm exposure is purely photothermal, the comparison with the current analysis of 413-nm 0.1 s and 1.0 s A/E_a_ values is valid. In fact, both the current simulated 413-nm and the previously measured 2-µm thermal profiles are considered equivalent MVL thermal responses, and thus comparable. In the 2-µm paper, the Ω_PT_ values were corrected by scaling the thermal profiles by less than 10% at each time point, supporting a concept that small changes in thermal profiles can exaggerate the value of Ω. This view of minimal thermal differences impacting Ω_PT_ supports our CDI model as being accurate.

Due to irradiance reciprocity for the 100-200-s exposures, their temperature rises also show reciprocity (**Table 1**). This may account for the well-behaved prediction of the photochemical A/E_a_ values in Ω_PC_. It is interesting that, although the A/E_a_ values for the two processes are similar in magnitude, the effect of the low overall temperatures for the purely photochemical process was opposite for the Ω_PC_ (one) and Ω_PT_ (zero). This again supports the CDI model. The question of temperature dependence of the photochemical processes remains elusive. We have empirical data showing that purely photochemical damage in our artificially pigmented *in vitro* retinal model is accelerated at temperatures as high as 46.1 °C (11.6 °C temperature rise) (33). This temperature is below the simulated value for the 40 s and 60 s 413-nm exposures (**Table 1**). We continue to investigate the thermal contributions to photochemical damage mechanisms in an effort to identify the temperature at which purely photochemical processes are inactivated to support the ongoing hypothesis described in **Figure 1**. In general, our results reinforce the need to measure or simulate thermal responses to exposures consistent with purely photochemical damage mechanisms in order to characterize the process. Having thermal responses may provide a favorable addition to the widely used irradiance reciprocity qualification for purely photochemical damage processes.

Finally, the power of the $\phi_{\tau }$ photochemical switch is shown in **Figure 6**. Delivery rate of photons appears to be a fundamental biochemical requirement for driving damage processes in pigmented cells. The use of threshold average $\phi_{\tau }$ resolves the nonlinearity between damage outcome and delivery of radiant exposure (**Figure 2A**) described above. This concept agrees with the expectation that irradiance drives both thermal responses and the production of RXS. From **Figure 6B**, it would seem that this principle extends to the nonhuman primate model, and thus to humans. Regardless of a universal switch value of $\phi_{\tau }$ , the underlying tenet for a photon flux threshold remains important for predicting retinal health when exposed to visible lasers.

## Data Availability

The original contributions presented in the study are included in the article/supplementary material, further inquiries can be directed to the corresponding author/s.
